# Assessing the impact of disabilities on healthcare access among Sudanese refugees in Egypt during the 2023 crisis: a discriminant analysis approach

**DOI:** 10.3389/fmed.2025.1646347

**Published:** 2025-12-12

**Authors:** Hatem E. Semary, Suzan Abdel-Rahman, Khamis A. Al-Karawi, Mahmoud M. Abdelwahab, Komla Mawunyo Dossouvi, Amr Elkelish, Abdelhamid El Shabrawy

**Affiliations:** 1 Department of Mathematics and Statistics, College of Science, Imam Mohammad Ibn Saud Islamic University (IMSIU), Riyadh, Saudi Arabia; 2 Faculty of Graduate Studies for Statistical Research, Cairo University, Cairo, Egypt; 3 School of Science, Engineering, and Environment, Salford University, Great Manchester, United Kingdom; 4 Department of Microbiology, Global Health Research Institute, Lomé, Togo; 5 Department of Biology, College of Science, Imam Mohammad Ibn Saud Islamic University (IMSIU), Riyadh, Saudi Arabia; 6 Faculty of Graduate Studies for Statistical Research, Cairo University, Cairo, Egypt

**Keywords:** Sudanese refugees, disabilities, healthcare access, living conditions, discriminant analysis

## Abstract

**Introduction:**

The longstanding presence of Sudanese refugees in Egypt—fueled by decades of political instability, conflict, and economic hardship in Sudan—has evolved with the recent 2023 crisis. This study investigates the living conditions, health status, and the impact of disabilities on healthcare access among newly displaced Sudanese in Egypt.

**Methods:**

A phone-based survey was administered to 531 Sudanese refugees. Descriptive statistics were first used to summarize the key demographic and household characteristics of the sample. The data was then analyzed using discriminant analysis to distinguish between families with and without healthcare challenges.

**Results:**

The sample had a mean age of 39.85 years, and the majority were female (75.5%), married (61.4%), and possessed a university-level education or higher (50.5%). On average, 4.92 family members migrated to Egypt with the respondent. The analysis revealed that chronic conditions such as spinal diseases and diabetes, along with the need for regular monthly treatment, were the strongest predictors of healthcare challenges among Sudanese refugee families. Additionally, disabilities including vision impairments, mobility difficulties, and intellectual disabilities were significantly associated with increased barriers to accessing healthcare services.

**Discussion:**

These findings underscore the compounded vulnerabilities of Sudanese refugees with disabilities and highlight the urgent need for targeted interventions to enhance healthcare accessibility and overall wellbeing. The results further stress the need for disability-sensitive health responses during refugee crises.

## 1 Introduction

Sudan has experienced many challenges, including a two-decade civil war and the 2003 Darfur genocide. These events have caused the country to face severe humanitarian crises marked by widespread hunger, famine episodes, and the displacement of an astounding number of its citizens. These tragic events have contributed to the loss of over two million lives (1). For many years, the status of Sudanese refugees in Egypt has been a significant concern. Of Sudan’s neighbors, Egypt has the second-highest number of asylum seekers after Chad. As of September 11, 2023, there have been 310,000 Sudanese refugees arriving in Egypt since April 15, 2023. More pressure is created by the fact that, in addition to the recent arrivals from Sudan, the majority of Sudanese who were living in Egypt before the crisis have asked to stay because they are unable to return because of the ongoing violence and instability in their nation (2).

Around 88% of registered new arrivals are from Khartoum. Furthermore, 22% of registered individuals suffer from at least one challenge: lack of legal documentation, at-risk or separated and unaccompanied children, single parents, people with disabilities, and people having severe health conditions. The illegal entry of Sudanese through smuggling due to their lack of valid passports and entry visas to Egypt exposes many Sudanese to the risk of detention and deportation. Among registered new arrivals, 46% reported entering illegally. Nearly two out of every three refugees live in Giza; one in three in Cairo, about 8% live in Alexandria, and 2% are distributed among other regions (2).

Many studies have examined the circumstances of displaced Sudanese to neighboring countries, focusing on Darfur refugees. Lozan and Hasan (3) discussed the mental health challenges faced by Sudanese refugees, emphasizing their experiences, including war, food insecurity, ethnic conflicts, and seeking better socio-economic conditions (4). Meffert and Marmar (2) investigated the psychological health of Darfur refugees in Cairo and found that a considerable portion was grappling with profound psychological turmoil, including signs of depression and enduring trauma, with individuals involved in both violent confrontations in their homes and community (5). Mahmoud focused on how Sudanese refugees’ lives change due to forced migration, highlighting their experiences of displacement and asylum-seeking in Egypt (6) Kunna (7) also focused on Darfur refugees in Egypt, shedding light on their poor situations characterized by lacking basic services and facing discrimination and mistreatment by authorities (3). The study also indicated that these refugees face challenges of integration and identity, often experiencing a dual identity crisis and feeling alienated from both Egyptian and Sudanese communities (6). Ibreck and Seeka (8) studied the South Sudanese refugees in Cairo to illustrate the refugees’ perspectives, assessing their political actions and reactions toward humanitarian governance (9). Hamdy (10) explored the quality of life and health-seeking behavior among Sudanese immigrants in Egypt, indicating that post-war Sudanese immigrants had diverse perceptions of overall quality of life. Most immigrants exhibited positive health-seeking behavior (11).

Studies have addressed the effects of the devastating conflict in Sudan. According to Matlin et al., (5), refugees face multiple health issues, such as inadequate access to services, stigma and discrimination, mental health problems, administrative and legal barriers, and neglect of their needs (12). Shafib et al. (13) also indicated that extreme poverty, illiteracy, low mental health insurance coverage, and unequal access to services negatively impact mental health outcomes, leading to increased prevalence of depression and post-traumatic stress disorder (14). Aderinto and Olatunji (1) confirmed that the conflict negatively affected the health and wellbeing of the population, with women and children being the most affected (15).

Considering the recent Sudan crisis in 2023, there have been limited studies addressing the challenges faced by displaced Sudanese refugees in Egypt, particularly regarding access to formal employment, education, and basic healthcare. Refugees with chronic diseases or disabilities encounter significant difficulties in obtaining necessary healthcare due to societal changes, financial constraints, and a lack of support programs. Therefore, it is essential to assess the availability of healthcare services for these individuals and identify their specific challenges.

To emphasize the importance of this study and clarify the research gap compared to previous studies, most studies have focused primarily on the social and psychological conditions of Sudanese refugees affected by conflicts in previous years, especially those coming from Darfur. These studies covered multiple aspects such as mental health, legal and economic challenges as addressed by Grabska (4) and Iezzoni and Long-Bellil (16), identity crises of refugees (10, 17, 18), as well as details of the lives of Sudanese refugees in Egyptian society, including their living conditions and lack of basic services (3, 9). Our study distinguishes from previous studies in that it focuses on Sudanese refugees’ health and living conditions and highlights the specific challenges these refugees face in accessing health care. We addressed the impact of disability and chronic diseases on these individuals’ opportunities to access necessary health care. Identifying the factors influencing their access to essential healthcare services can inform recommendations to improve their health conditions in Egypt.

## 2 Materials and methods

### 2.1 Data source

Data were collected from a sample of Sudanese who came to Egypt after the 2023 crisis. The data were collected through phone interviews, in which respondents were accessed through their Egyptian mobile phones. The phone numbers were selected from a database built by the Egyptian Center for Public Opinion Research (Baseera). Baseera has been building its database from areas in Greater Cairo where Sudanese are concentrated. The survey questionnaire (Supplementary File 1) was designed to evaluate the circumstances of displaced Sudanese in Egypt following the crisis in Sudan. Sudanese residing in Egypt who are in Baseera’s survey database were called upon to collect information about new arrivals to Egypt after April 15. The sample was collected to get estimates with a 95% significance level at the national level, with a margin of error of < 5% and an effect size of more than 0.5, resulting in a sample of 531 Sudanese.

### 2.3 Data collection tool and studied variables

The survey aims to assess the living conditions and assistance the Sudanese entrants receive in Egypt. The survey questionnaire is designed to evaluate the circumstances of displaced Sudanese in Egypt following the crisis in Sudan. It gathers information about the migrants’ ages, gender, educational status, and marital status. The form inquiries about the migration circumstances, the way of entry into Egypt, and the number of family members who have arrived in Egypt while also addressing those left behind in Sudan. The form also delves deeply into health status, inquiring about any chronic diseases or disabilities they may have. Additionally, it details their living conditions and employment status, reviews the various forms of aid the migrants have received, and elucidates the primary challenges they face in Egypt. The survey includes different types of questions, such as closed-ended, open-ended, multiple-choice, and Likert scale, and takes around 10 min to complete.

### 2.4 Statistical methods

The study utilized various statistical methods, including descriptive statistics, to calculate means, standard deviations, and frequency distributions for analyzing demographic characteristics. We provided summary statistics of the baseline characteristics of Sudanese refugees, detailing their migration conditions and focusing on family members’ health conditions. Additionally, we outlined the living conditions of Sudanese refugees in Egypt, highlighting the assistance they received and the challenges they faced.

The study utilized discriminant analysis to assess health-related factors affecting families with and without healthcare problems. The dependent variable—presence or absence of a healthcare problem—was derived from responses to the question: “Is your family currently experiencing any problems in accessing required healthcare services in Egypt?” Households that answered “Yes” to any difficulty related to consulting a doctor, receiving treatment, obtaining medication, or continuing follow-up care were coded as 1, indicating the presence of a healthcare problem. Those who answered “No” were coded as 0, indicating no healthcare problem.

Discriminant analysis is a valuable technique for predicting the likelihood of outcomes across two or more categories. It generates a discriminant function incorporating several independent variables (discriminator variables). This function helps differentiate between observations that belong to each category of the outcome variable. The discriminant analysis focuses on assessing the distance between centroids to distinguish between different outcome categories effectively. One key assumption of this method is that the discriminator variables must be at least on an interval scale.

In contrast, binary logistic regression allows independent variables to be of any measurement scale. Another critical difference is that in a binary logistic model, the outcome variable must be dichotomous (having only two categories). At the same time, discriminant analysis can handle an outcome variable with multiple categories. In our study, we only developed one discriminant function because the outcome variable has two categories (No. of discriminant functions = No. of groups – 1).

## 3 Results

### 3.1 Baseline characteristics

Data was collected from 531 Sudanese refugees aged 18 years and over who came to Egypt after April,15, 2023. The mean age was 39.85, with a 13.19 standard deviation. The majority were female (75.5%), married (61.4%), and with a university education or higher (50.5%). The average number of family members who came to Egypt, including the respondent, after the events of April was 4.92 ± 3.07. Respondents indicated that the average number of working-age males in their families who came to Egypt is 0.84 and females aged (15–49) is 1.64 on average, while the average number of children aged 2 years to < 6 is 0.53 and rise to 0.80 of children aged 6 years to < 13 years, as shown in Table 1.

**TABLE 1:** Summary statistics of baseline characteristics of Sudanese refugees.

Baseline characteristics	Labels	N (%)
Age (Mean ± SD)	39.85 ± 13.19	
Gender	Male	130 (24.5)
	Female	401 (75.5)
Educational level	Less than secondary	71 (13.4)
	Secondary and above intermediate	192 (36.2)
	University and above	268 (50.5)
Marital status	Never married	124 (23.4)
	Married	326 (61.4)
	Widowed/divorced	81 (15.3)
Household head	Yes	403 (75.9)
	No	128 (24.1)
Status of other family members	All of them came to Egypt	163 (30.7)
	I had to left some of them	363 (68.4)
	Some of them traveled to other countries	(0.9)
Composition of family members came to Egypt (Average)	Children under 2 years	0.84 ± 1.03
	Children 2-< 6 years	1.64 ± 1.36
	Children 6-< 13	0.21 ± 0.508
	Children 13-under 18)	0.53 ± 0.86
	Working-age males (15–64)	0.80 ± 1.17
	Females (15–49)	0.36 ± 0.74
	Elderly people (60 years and over)	0.39 ± 0.67
Family members left behind in Sudan	Males	324 (89.3)
	Females	139 (38.3)
	Children	90 (24.8)
	Elderly	100 (27.5)
Average number of family members left in Sudan	Number of males	1.86 ± 1.34
	Number of females	2.38 ± 1.66
	Number of children	2.70 ± 2.15
	Number of elderly	1.38 ± 0.632
Total	531	

Of the Sudanese refugees who left their family members in Sudan, 80.9% left behind their male family members, 38.3% left female members, and only 248.% left children. The average number of male members left in Sudan is 1.86 ± 1.34 and increased to 2.70 ± 2.15 for children. 62% of Sudanese refugees explained that they left their family members due to the difficulty of obtaining a visa, as presented in Table 1.

### 3.2 Migration conditions

Half of the Sudanese refugees entered through the Qustul border crossing, while 45% crossed through the Arqin border crossing and 5% used other crossings. More than half needed a visa to come to Egypt (55.6%), and most of them had valid passports (84.6%) and were accommodated on the passport (54.2%).

The reasons for Sudanese choosing to seek refuge in Egypt are the proximity of the distance (51.8%), the ease of entry procedures (5.3%), the presence of relatives (5.6), and others (27.5%). More than 95% of the refugees intend to reside in Egypt until the situation in Sudan stabilizes, and 63% expect the remaining relatives in Sudan to come to Egypt soon, presented in Supplementary Table 1.

### 3.3 Health conditions

Most refugees (68.7%) indicated that one of their family members suffers from at least one chronic disease. About 41.1% mentioned high blood pressure, close to third mentioned allergies (32.0%), diabetes (33.0%), and joint Stiffness (34.7%), while the lowest percentage (11.1%) reported heart diseases.

Also, 11.1% of Sudanese refugees reported that one of their family members suffers from a movement disability, and 11.1% mentioned a vision impairment. About 20.2% of respondents reported that at least one family member currently living with them in Egypt suffers from anemia or malnutrition, and 63% confirmed that a household member needs regular monthly treatment, presented in Supplementary Table 2.

### 3.4 Living conditions

About 54% of the Sudanese refugees live in rented apartments, and 42.4% live with their relatives and friends. Also, 23.5% stated that they share the apartment with other Sudanese families, while 23% live alone. More than half of the Sudanese refugees had relatives and acquaintances in Egypt before the recent Sudanese crisis, while 42% did not.

62.7% of Sudanese refugees reported working in Sudan, and only 4.7% were unemployed and looking for work. But after the crisis, more than a third (33.7%) became unemployed and looking for work, 35.6% intend to work, and only 8.5% are currently employed. In addition, 94.9% of the refugees explained that no family member works. At the same time, 39.9% reported that subsidies are the primary source of family and food expenditures, while others depend on money saved from Sudan to cover family (35%) and food expenses (59.5%). The refugees indicated that they spend most on our food (79.7%), housing rent (30.3%), health care (29%), and transportation (4.1%), presented in Supplementary Table 3.

### 3.5 Aid and challenges facing Sudanese refugees

**FIGURE 1:**
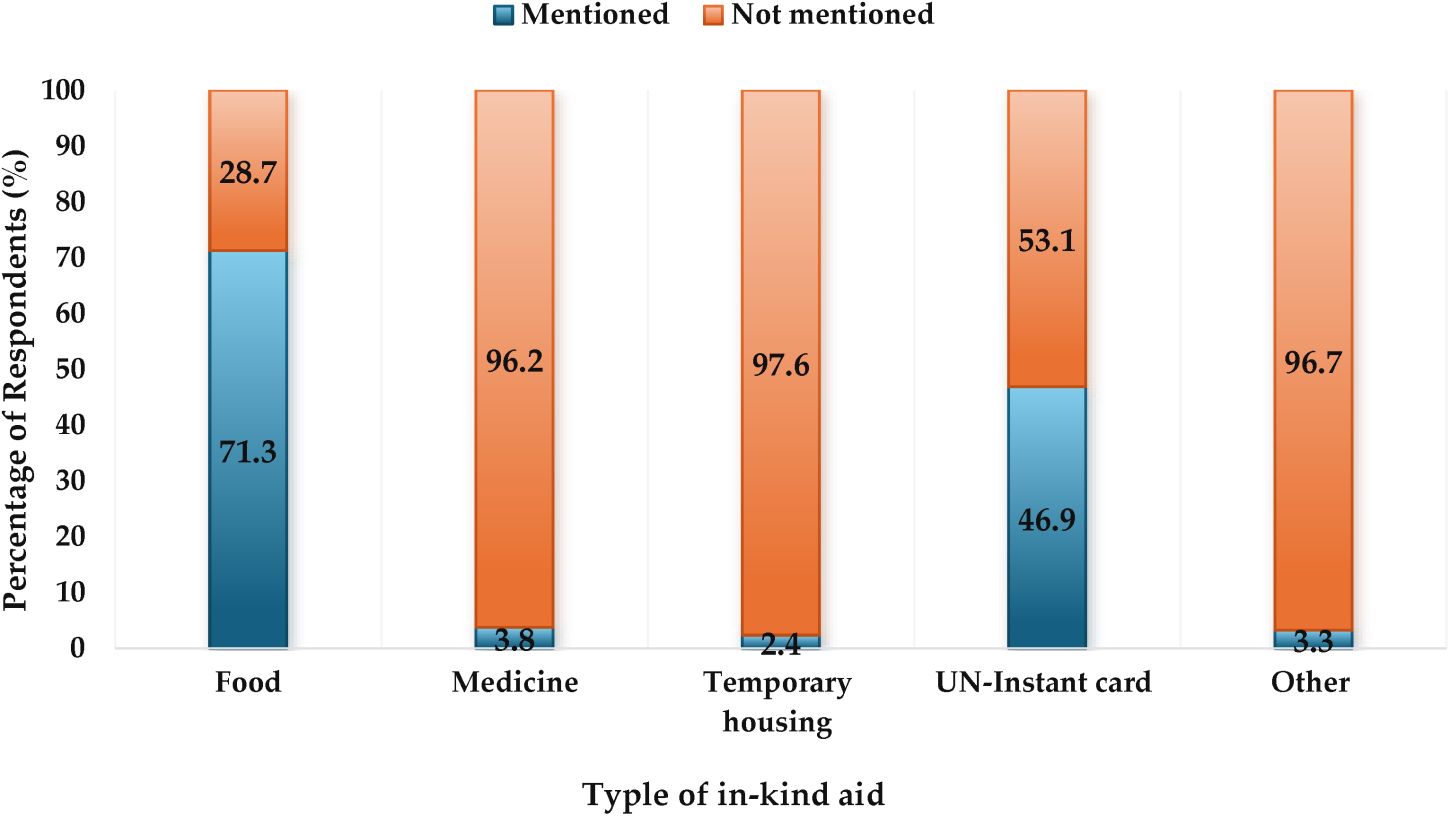
Types of in-kind aid Sudanese refugees received after coming to Egypt.

Sudanese refugees are receiving various types of aid: 33.1% receive financial support, while 23.5% benefit from in-kind resource. There were also 15.8% (84) who received financial and in-kind aid, as shown in Supplementary Table 4. Unfortunately, approximately 27.5% (146) do not receive assistance. Figure 1 details the types of in-kind aid received by Sudanese refugees after coming to Egypt. The percentages reflect the proportion of respondents who mentioned receiving each type of aid out of the total sample. The highest reported aid type is food at 71.3%, while the least reported are medicine and temporary housing at 3.8 and 2.4%, respectively. The total value of the financial aid provided to each family did not exceed L.E 2335.

**FIGURE 2:**
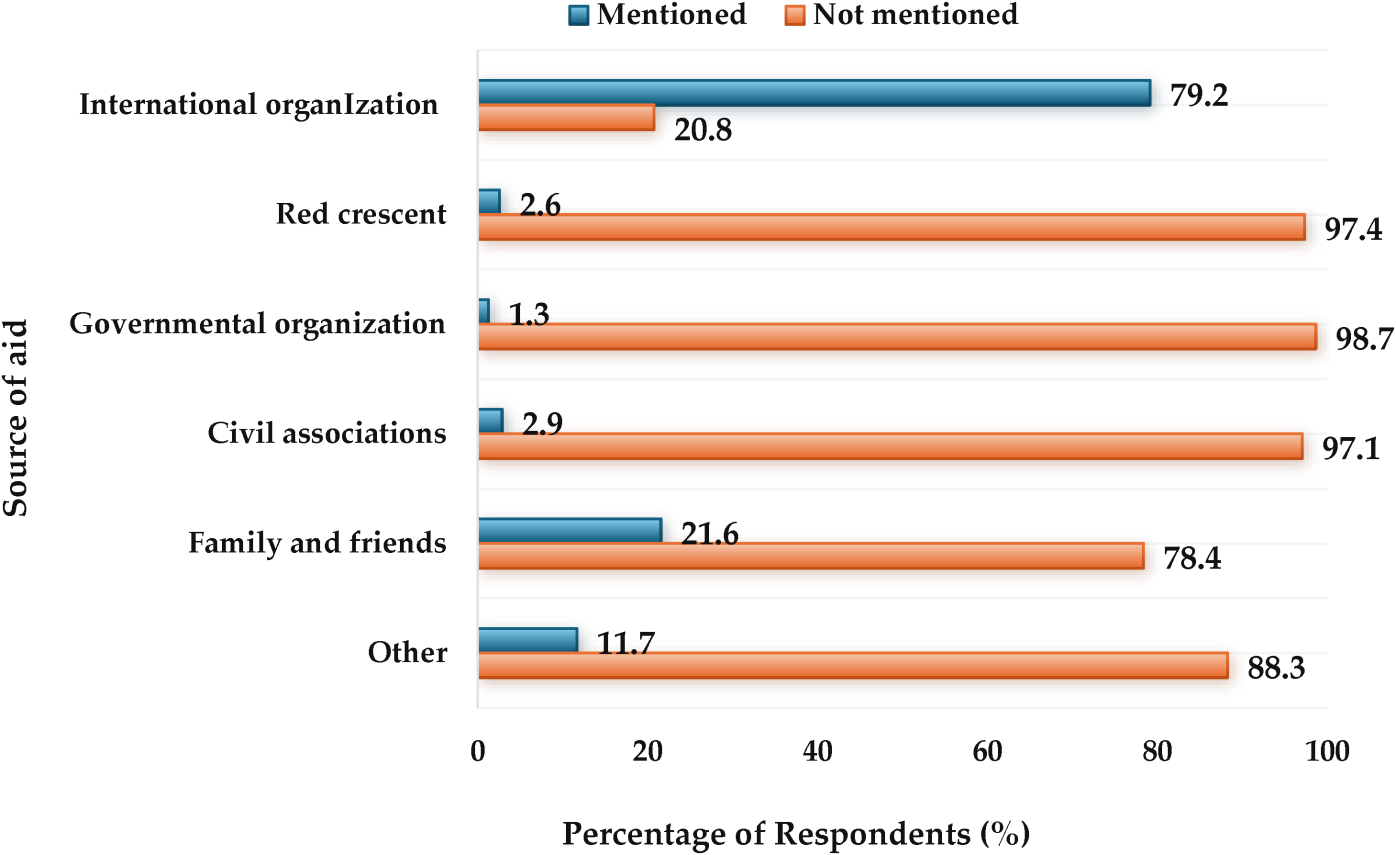
The source of aid received by Sudanese refugees.

Figure 2 displays the sources of aid received by Sudanese refugees. The percentages represent the proportion of respondents who reported receiving aid from each source out of the total sample. Notably, a large majority, 79.2%, reported receiving assistance from UN-affiliated international organizations, highlighting their significant role in the aid effort. In contrast, contributions from the Red Crescent, governmental organizations, and civil associations were minimal, each below 3%. Aid from family and friends accounted for 21.6%. When asked whether this aid covers their necessary needs, only 5.2% of respondents found the aid sufficient, while 43.3% found it insufficient. Nearly half (49.2%) did not receive an instant card from WFP or UNICEF. Additionally, most respondents (71.4%) were unfamiliar with e-wallet applications, with only 28.6% aware of them.

**FIGURE 3:**
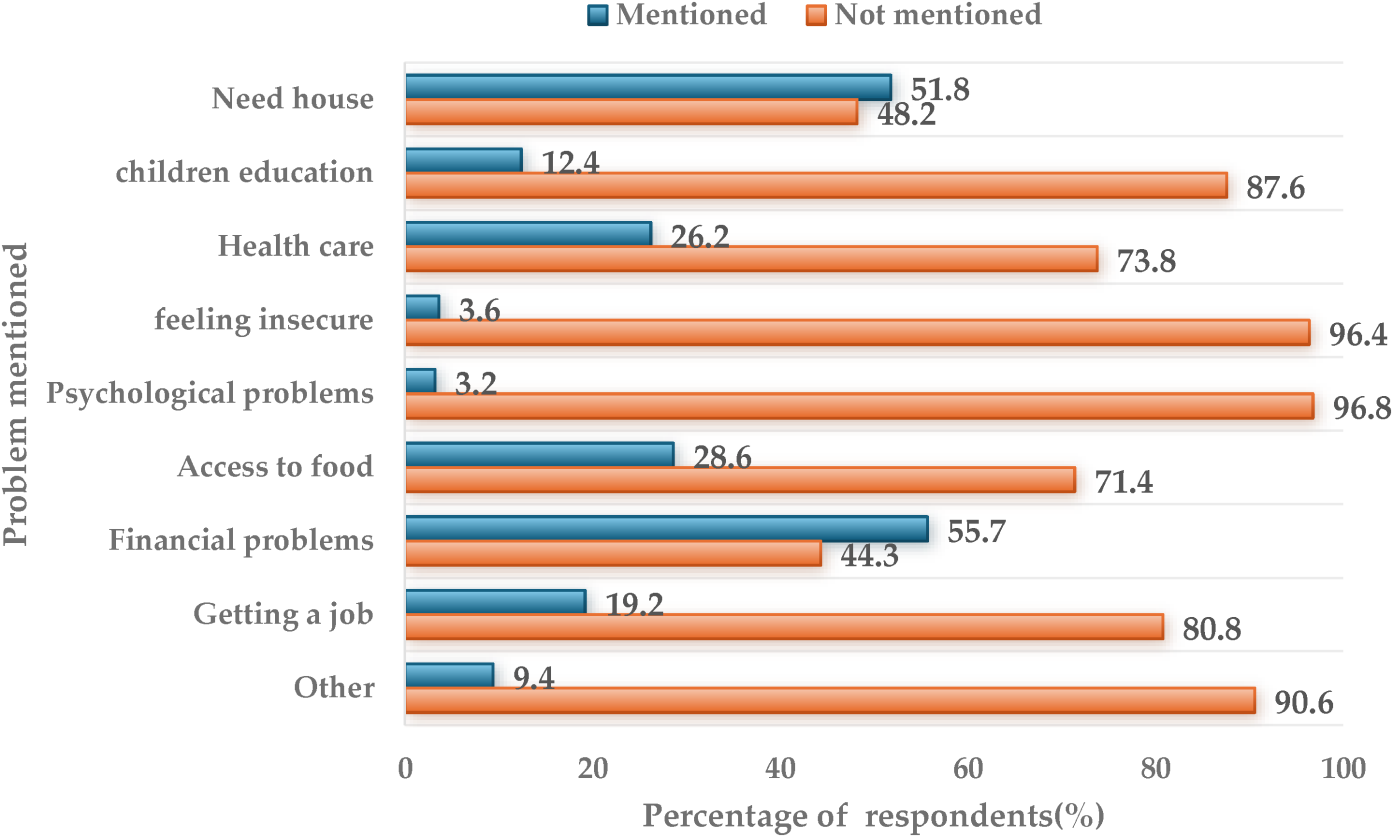
The most critical problems faced by Sudanese refugees in Egypt.

Figure 3 illustrates the most critical problems faced by Sudanese refugees in Egypt. The percentages reflect the proportion of respondents who identified each issue as a major challenge, based on the total sample. According to the survey, financial problems (55.7%) and housing (51.8%) are the most frequently reported problems. Additionally, access to food (28.6%) and healthcare (26.2%) are also significant problems. Employment is a concern for 19.2% of respondents, while children’s education was mentioned by 12.4%. Notably, insecurity and psychological problems were the least reported, at 3.6 and 3.2%, respectively, suggesting these are less prevalent compared to material hardships and basic survival needs.

The discriminant analysis model in this study examined various health-related factors, including the number of family members needing regular monthly treatment and those with specific disabilities or chronic conditions. Notably, three disability variables—intellectual disability, vision disability, and movement difficulties—significantly impacted the discrimination between families with and without health services problems. Among these, vision disability had the most potent effect, followed by movement difficulties, while intellectual disability had the least impact.

Table 2 presents the mean and standard deviation for the existence of healthcare problems across 11 discriminator variables, categorized into two groups: “No, there is no healthcare problem” and “Yes, there is a healthcare problem.” Notable differences are observed between the means of the dependent variable in these two categories. For instance, the number of family members needing regular monthly treatment averages 0.83 in the “No” category and 1.42 in the “Yes” category. Similarly, the averages for diabetes are 0.34 (“No”) versus 0.67 (“Yes”), for allergies 0.35 (“No”) versus 0.63 (“Yes”), and for spine diseases 0.20 (“No”) versus 0.44 (“Yes”). These four variables are identified as effective discriminators between the presence and absence of healthcare problems.

**TABLE 2:** Mean and standard deviation of healthcare problems across discriminator variables.

Existence of health care problems	Discriminator variables (numbers)	Mean	SD
No	Family members need monthly treatment	0.83	0.908
	Family members with allergies	0.35	0.678
	Family members with intellectual disabilities	0.04	0.199
	Family members with vision disabilities	0.13	0.448
	Family members with diabetes	0.34	0.631
	Family members with high blood pressure	0.46	0.685
	Family members with spine diseases	0.20	0.476
	Family members with difficulties or disabilities in movement	0.08	0.280
	Family members with joint stiffness diseases	0.34	0.525
	Family members with other diseases	0.20	0.544
Yes	Family members need monthly treatment	1.42	1.129
	Family members with allergies	0.63	0.918
	Family members with intellectual disabilities	0.07	0.310
	Family members with vision disabilities	0.27	0.769
	Family members have diabetes	0.67	0.793
	Family members with high blood pressure	0.76	0.952
	Family members with heart diseases	0.19	0.427
	Family members with spine diseases	0.44	0.615
	Family members has difficulties or disabilities in movement	0.14	0.427
	Family members with joint stiffness diseases	0.53	0.695
	Family members with other diseases	0.37	0.593
Total	Family members need monthly treatment	0.99	1.003
	Family members with allergies	0.43	0.757
	Family members with Intellectual disabilities	0.05	0.234
	Family members with vision disabilities	0.17	0.553
	Family members with diabetes	0.42	0.692
	Family members with high blood pressure	0.54	0.774
	Family members with heart diseases	0.12	0.339
	Family members with spine diseases	0.26	0.526
	Family members with difficulties or disabilities in movement	0.10	0.325
	Family members with joint stiffness diseases	0.39	0.580
	Family members with other diseases	0.24	0.562

Table 3 indicates that all discriminator variables are statistically significant in differentiating between the two categories of the outcome variable related to healthcare problems. This table tests the null hypothesis (H_0_) that the means of all discriminator variables are equal across the groups concerning the existence of healthcare problems. In contrast, the alternative hypothesis (H_1_) posits that these means are unequal across the groups. The findings support rejecting the null hypothesis, suggesting meaningful differences in the discriminator variables. It is clear that all discriminator variables are significant; indicating that the variable succeeded in discriminating between observations that have an existence of health care problems and other observations which have no existence of health care problems.

**TABLE 3:** Tests of equality of group means.

Discriminators: numbers of	Wilks’ Lambda	*F*	df1	df2	Sig.
Family members need monthly treatment	0.935	37.057	1	529	0.000
Family members with allergies	0.974	14.224	1	529	0.000
Family members with intellectual disabilities	0.995	2.475	1	529	0.016
Family members with vision disabilities	0.987	6.996	1	529	0.009
Family members with diabetes	0.955	24.740	1	529	0.000
Family members with high blood pressure	0.971	15.922	1	529	0.000
Family members with heart diseases	0.985	8.225	1	529	0.004
Family members with spine diseases	0.960	22.197	1	529	0.000
Family members with difficulties or disabilities in movement	0.992	4.095	1	529	0.04
Family members with joint stiffness diseases	0.980	10.747	1	529	0.001
Family members with other diseases	0.981	10.422	1	529	0.001

The goodness-of-fit indicators of the discriminant analysis model suggest that it is statistically acceptable. The model explained approximately 10.4% of the variance in the outcome variable (the existence of healthcare problems), based on a canonical correlation of 0.324, which reflects a modest relationship between the predictor variables and the grouping outcome.

Table 4 provides the classification results. The model correctly classified 68.4% of the original grouped cases, with 281 out of 392 cases (71.7%) correctly classified in the “No healthcare problem” group, and 82 out of 139 cases (59%) correctly classified in the “Yes healthcare problem” group. Cross-validation using the leave-one-out method yielded a similar correct classification rate of 66.3%, which supports the stability of the model, though its predictive performance remains moderate and below the majority-class baseline (73.8%).

**TABLE 4:** Corrected classification measures.

Dependent variable	Predicted group membership		Total
	No, there is health care problem	Yes, there is health care problem	
**Original**			
No, there is health care problem	281	111	392
Yes, there is health care problem	57	82	139
No, there is health care problem%	71.7	28.3	100.0
Yes, there is health care problem%	41.0	59.0	100.0
**Cross-validated**			
No, there is health care problem	275	117	392
Yes, there is health care problem	62	77	139
No, there is health care problem%	70.2	29.8	100.0
Yes, there is health care problem%	44.6	55.4	100.0

The proportion of variance explained and the correct classification rate indicate that the discriminant function is statistically valid and suitable for identifying key variables associated with healthcare problems, despite its limited predictive accuracy.

Table 5 presents the standardized and unstandardized discriminant function coefficients for all discriminator variables. The standardized coefficients allow for comparison of the relative importance of each variable in distinguishing between families with and without healthcare problems. According to reported standardized values, the most important variable is the number of family members with spine diseases (0.349), followed by those with diabetes (0.320), and then those who need regular monthly treatment (0.312). In contrast, the variable with the least relative importance is the number of family members with high blood pressure (0.020).

**TABLE 5:** Standardized and unstandardized canonical discriminant function coefficients.

Discriminators: numbers of	Standardized coefficients	Unstandardized coefficients
Family members need monthly treatment	0.312	0.321
Family members with allergies	0.113	0.151
Family members with intellectual disabilities	0.191	0.818
Family members with vision disabilities	0.219	0.399
Family members with diabetes	0.320	0.473
Family members with high blood pressure	0.020	0.027
Family members with heart diseases	0.117	0.349
Family members with spine diseases	0.349	0.677
Family members with difficulties or disabilities in movement	0.114	0.352
Family members with joint stiffness diseases	–0.051	–0.089
Family members with other diseases	0.298	0.535
Constant	–	–1.047

DF = 0.321* No. of family members who need monthly treatment, take it regularly + 0.151* No. of family members has Allergy + 818* No. of family members has Intellectual disability + 0.399* No. of family members has vision disability + 0.473* No. of family members has diabetes + 0.027* No. of family members has high blood pressure + 0.349* No. of family members has heart diseases + 0.677*No. of family members has spine diseases + 0.352*No. of family members has movement disability –0.089* No. of family members has Joint stiffness diseases + 0.535*No. of family members has other diseases –1.047.

On the other hand, the unstandardized discriminant function coefficients are used to construct the discriminant function equation. These coefficients reflect the partial contribution of each variable to the discriminant score, expressed in the original units of measurement. However, they are not suitable for assessing the relative importance of variables due to differences in measurement scale. According to the unstandardized coefficients, the number of family members with intellectual disabilities has the highest coefficient (0.818), where each additional individual with intellectual disabilities increases the discriminant score by 0.818 units, holding other variables constant. Additionally, the number of family members with spine diseases and other chronic conditions also have relatively large unstandardized coefficients (0.677 and 0.535, respectively), contributing more to the discriminant score. In contrast, the coefficient for high blood pressure (0.027) indicates a small contribution to the discriminant function.

## 4 Discussion

Following the 2023 disturbances that rocked Sudan, a fresh group of refugees have sought safety inside Egypt’s boundaries. Sudanese refugees represent one of the largest refugee populations in Egypt, which highlights the importance of carefully examining their health status and medical needs. The health conditions of displaced Sudanese refugees in Egypt are a major concern, given their limited access to adequate healthcare services. According to statistics, an estimated 250,000 Sudanese refugees have sought refuge in Egypt, facing significant challenges in accessing healthcare facilities.

The health of Sudanese refugees is influenced by various social and economic factors, which often create barriers to accessing health services. This study explores the multiple challenges they face, focusing on their living conditions and health problems. Additionally, it examines factors that explain reported healthcare issues by shedding light on their overall health status.

The ongoing armed conflict in Sudan has had devastating effects on public health, exacerbating existing challenges and creating new ones. The conflict has led to heavy fighting, civilian casualties, and a lack of medical services in hospitals. The conflict also has significant impacts on mental health, including post-traumatic stress disorder, depression, and anxiety, and increases the prevalence of conflict-related sexual violence (1, 2).

Several studies addressed the marginalization of Sudanese refugees in Cairo, highlighting their struggle to access healthcare and legal rights, which negatively impacts their health outcomes (4, 5, 19). Consistent with previous studies, this study found that Sudanese refugees suffer from significant health and living conditions. Considerable percentage suffering from chronic diseases such as high blood pressure, diabetes, and joint stiffness. Their living conditions varied; some rent apartments, while others live with relatives. Employment status changed after the crisis, with many losing their jobs. Many families rely on financial support, with food expenses being their primary concern.

Most aid comes from international organizations; however, many refugees found this assistance insufficient, and some did not receive any support. In addition, many faced difficulties in dealing with electronic payment options, as a sizable percentage of them are unfamiliar with e-wallet applications.

Our findings indicated that the most reported problems were housing, food, and access to healthcare, while psychological issues and feelings of insecurity were among the least mentioned. This was unexpected, as previous studies have shown that many refugees suffer from both physical and mental health challenges due to displacement and the trauma they have experienced (3, 19). One possible explanation is that participants were primarily focused on urgent survival needs—such as food, shelter, and healthcare—given the difficult circumstances they face. These immediate concerns may have overshadowed mental health issues. Additionally, the underreporting of psychological problems could be related to social stigma or limited awareness of mental health among the refugee population.

Regarding healthcare problems, the standardized discriminant coefficients indicated that the most influential variables in distinguishing between families with and without healthcare problems were spine diseases, followed by diabetes, and the need for regular monthly treatment. Although intellectual disabilities contributed to the model, their relative influence was smaller compared to the leading factors.

Among the contributing variables, visual impairment stood out as a notable factor influencing healthcare access. Individuals with visual disabilities face several challenges, including limited access to information, which is often not available in accessible formats such as braille, audio recordings or large printed information. Additionally, physical barriers such as inadequate transportation and lack of accessible infrastructure in health facilities, further hinder access to care (9, 11, 17). Global and regional studies have reported similar findings, showing that individuals with disabilities generally experience poorer access to healthcare services compared to those without disabilities. For example, a study in the United States showed that people with visual impairments are more likely to experience problems in receiving the necessary medical care (17).

Similarly, individuals with hearing impairments may experience communication challenges with caregivers due to a lack of sign language interpreters. Architectural barriers and transportation difficulties also hinder access for those with physical disabilities. Additionally, individuals with cognitive or intellectual disabilities may struggle to understand and retain medical instructions. There are also several complex factors, including environmental, social, and economic barriers, that influence access to healthcare for people with disabilities. People with disabilities often suffer from limited educational and employment opportunities, which increases their overall vulnerability and poor health. Spatial barriers, such as lack of adequate sanitation facilities and discrimination and negative beliefs about them, are major factors that hinder their access to care. Feelings of shame and communication difficulties may also lead them not to express their health needs, which leads to their health conditions worsening. Furthermore, high costs and transportation challenges pose additional barriers, making people with disabilities more vulnerable to health neglect (11, 12, 14–16).

Previous studies have shown that perceptions of quality of life among refugees are influenced by gender, education, and financial status. While many immigrants demonstrated positive health-seeking behavior, barriers such as limited awareness of services, competing priorities, insufficient financial resources, and long waiting times remain prevalent (10).

WHO has highlighted health issues faced by refugees and migrants across the Eastern Mediterranean region, including inadequate healthcare access, financial barriers, and psychological stress. Countries like Lebanon and Morocco report overwhelmed health systems, increased maternal and child mortality, and challenges in providing medical care. Initiatives such as public health insurance, community health programs, and psychosocial support exist but significant gaps in equitable access persist (18).

Egypt has made efforts to improve the situation of Sudanese refugees and meet their needs for education, health care, and other forms of protection. However, our results suggest that more efforts are needed to improve their living conditions and health conditions, especially for those who have suffered disabilities. Access to health care services must be enhanced, health awareness programs must be provided, and other efforts must be made to improve the health and quality of life of Sudanese individuals in Egypt. Increased international support and cooperation are essential to ensure the wellbeing and dignity of Sudanese refugees in their host country.

Our study emphasizes the urgent need for targeted interventions to address the challenges faced by displaced Sudanese refugees in Egypt. Facilitating access to healthcare services and livelihood opportunities will improve their situation and promote social integration. Moreover, the collaboration of government agencies, NGOs, and community-based organizations can ensure better support for this vulnerable population.

Regarding the study limitations, the study sample was drawn from the Baseera database of the Egyptian Center for Public Opinion Research, which contains contact details for Sudanese nationals residing primarily in Greater Cairo. This approach facilitated rapid data collection, particularly among newly displaced refugees. However, it may have introduced a degree of selection bias. The use of cellphone-based questionnaires likely excluded some of the most vulnerable segments of the population, such as undocumented refugees, individuals without mobile phones, and those residing in remote areas or informal settlements. Future research should consider employing a more representative sample to capture the experiences of marginalized refugees.

## 5 Conclusion

This study sheds light on the health and living conditions of Sudanese refugees in Egypt, who face challenges related to displacement, limited resources, food insecurity, and difficulties accessing healthcare. The most common problems reported were housing, food, and healthcare, while psychological issues and insecurity were among the least mentioned. Most refugees receive limited aid that does not fully meet their needs, and many are not using available digital tools like e-wallets. There is a need to improve basic services and ensure better access to support and healthcare.

The study also revealed a significant relationship between difficulties accessing healthcare and the presence of specific chronic diseases and disabilities among refugee households. Households with members suffering from spinal diseases, diabetes, and those requiring regular monthly medications were more likely to experience healthcare problems. Disabilities, particularly visual impairments, mobility impairments, and mental impairments, were also major factors limiting access to healthcare services. These findings highlight the need to prioritize support for households with these health conditions within aid and health programs to bridge the existing gap in healthcare access among the most vulnerable refugee groups.

## Data Availability

The original contributions presented in this study are included in this article/Supplementary material, further inquiries can be directed to the corresponding author.
