# Supplementary material for: Assessing the impact of disabilities on healthcare access among Sudanese refugees in Egypt during the 2023 crisis: a discriminant analysis approach
Source: Front Med (Lausanne). 2025 Dec 12;12:1646347. doi: 10.3389/fmed.2025.1646347 (PMC12741957; doi:10.3389/fmed.2025.1646347)
Supplement: Supplementary file 1 [file Supplementary_file_1.docx]

|  | 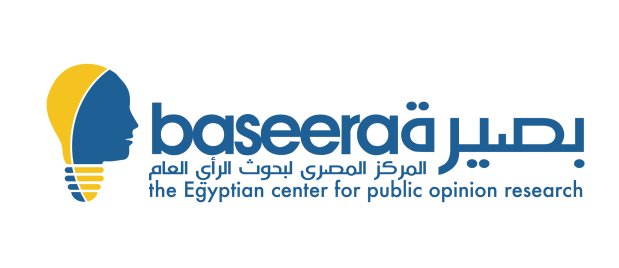 |
| --- | --- |

**Translated Questionnaire: Assessment of Sudanese Arrivals’ Conditions in Egypt Post-2023 Sudan Crisis**
**(Living Conditions and Assistance Survey)**
**July 2023**

**Introduction**
*Good morning/evening,*
*We are [INTERVIEWER NAME] from the Egyptian Center for Public Opinion Research (Baseera). We are conducting a survey in collaboration with UNICEF to assess the conditions of Sudanese arrivals in Egypt after the recent crisis in Sudan. The survey will take approximately 10 minutes. All data is confidential and will be used solely for research purposes.*

**Section 1: Demographic and Personal Information**

1. **Are you Sudanese?**
   - Yes
   - No (specify): __________
2. **Did you arrive in Egypt after the events of April 15, 2023?**
   - Yes
   - No
3. **What is your age (in full years)?**
   - Under 18
4. **What is your current governorate of residence in Egypt?**
5. **Do you reside in an urban or rural area?**
   - Urban
   - Rural
   - Don’t know
6. **Gender:**
   - Male
   - Female
7. **What is the highest educational level you have completed?**
   - Never attended school
   - Less than primary
   - Primary
   - Preparatory
   - Secondary
   - Post-secondary
   - University
   - Postgraduate
8. **Marital status:**
   - Never married
   - Married
   - Widowed
   - Divorced
   - Separated
9. **Are you the head of the household?**
   - Yes
   - No
10. **What is your relationship to the head of the household?**
    - Father
    - Mother
    - Husband
    - Wife
    - Son
    - Daughter
    - Other (specify): __________

**Section 2: Migration Conditions**

1. **Did all your family members come to Egypt, or did you have to leave some behind in Sudan?**
   - All came to Egypt
   - Had to leave some behind
   - Some traveled to other countries
2. **Family members left in Sudan:**
   - Males (number): ______
   - Females (number): ______
   - Children under 18 (number): ______
   - Elderly (60+ years) (number): ______
3. **Was leaving family members behind due to difficulty obtaining visas?**
   - Yes
   - No
4. **Total number of family members (including yourself) who came to Egypt after April:**
5. **Number of working-age males (15–64) in your family:**
6. **Number of females aged 15–49:**
7. **Number of children under 2 years:**
8. **Number of children aged 2–5 years:**
9. **Number of children aged 6–12 years:**
10. **Number of children aged 13–17 years:**
11. **Number of elderly (60+ years):**

**Section 3: Health Conditions**

1. **Does any family member have chronic diseases?**
   - Yes
   - No
2. **If yes, specify (number of individuals):**
   - Diabetes: ______
   - Hypertension: ______
   - Heart diseases: ______
   - Spinal diseases: ______
   - Joint stiffness/arthritis: ______
   - Allergies: ______
   - Other (specify): ______
3. **Does any family member have anemia or malnutrition?**
   - Yes (number): ______
   - No
4. **Does any family member require regular monthly treatment?**
   - Yes (number): ______
   - No

**Section 4: Living Conditions and Employment**

1. **Current type of housing in Egypt:**
   - Owned apartment
   - Rented apartment
   - Shared housing with other Sudanese families
   - Living with relatives/friends
   - Homeless
   - Other (specify): __________
2. **Primary source of household expenses:**
   - Income from work
   - Savings from Sudan
   - Remittances from abroad
   - Subsidies/aid
   - Other (specify): __________
3. **Primary source of food expenses:**
   - Cash purchases
   - Borrowing/credit
   - Bartering for food
   - Food aid
   - Other (specify): __________
4. **Employment status in Egypt:**
   - Currently employed
   - Unemployed and seeking work
   - Intend to work
   - Outside the labor force

**Section 5: Aid Received**

1. **Have you received any assistance (cash, in-kind, or services) in Egypt?**
   - Yes (cash)
   - Yes (in-kind)
   - Yes (both cash and in-kind)
   - No
2. **Types of in-kind aid received:**
   - Food
   - Medicine
   - Temporary housing
   - WFP/UNICEF instant card
   - Other (specify): __________
3. **Sources of assistance:**
   - UN-affiliated organizations
   - Red Crescent
   - Government agencies
   - NGOs
   - Family/friends
   - Other (specify): __________
4. **Is the assistance sufficient for your basic needs?**
   - Very sufficient
   - Sufficient
   - Insufficient
   - Completely insufficient
   - Don’t know

**Section 6: Challenges Faced**

1. **What are your most pressing challenges in Egypt?**
   - Housing
   - Financial problems
   - Access to food
   - Healthcare access
   - Employment
   - Children’s education
   - Safety/security
   - Psychological stress
   - Other (specify): __________

**Notes:**

- *Ethical approval for this survey was granted by the Research Ethics Committee of the Egyptian Center for Public Opinion Research (Baseera), Cairo, Egypt (Reference No. REC-Baseera/2023-07).*
- *Verbal consent was obtained from all participants.*

**Egyptian Center for Public Opinion Research (Baseera)**
*Tel: 33451392–33451395 | Fax: 33451392 | Website:*[*www.baseera.com.eg*](http://www.baseera.com.eg/)
