# Supplementary material for: Assessing the impact of disabilities on healthcare access among Sudanese refugees in Egypt during the 2023 crisis: a discriminant analysis approach
Source: Front Med (Lausanne). 2025 Dec 12;12:1646347. doi: 10.3389/fmed.2025.1646347 (PMC12741957; doi:10.3389/fmed.2025.1646347)
Supplement: Supplementary file 2 [file Supplementary_file_2.docx]

Table A-1: Migration conditions of Sudanese refugees

| **Migration conditions** | **Labels** | **N (%)** |
| --- | --- | --- |
| **Cross from Sudan to Egypt** | Arqin border crossing  Qustul border crossing  Other | 237 (44.6)  265 (49.9)  29 (5.5) |
| **Need visa to come Egypt** | Yes  No | 236 (44.4)  295 (55.6) |
| **Had valid passport** | Yes  No | 449 (84.6)  82 (15.2) |
| **Got accommodation on the passport** | Yes  No | 288 (54.2)  243 (45.8) |
| **Reasons for asylum in Egypt** | Close distance  Feeling of security and safety  Easy entry procedures  The presence of relatives residing in Egypt  others | 275 (51.8)  52 (9.8)  28 (5.3)  30 (5.6)  146 (27.5) |
| **Intention to stay in Egypt** | Yes  No | 505 (95.1)  26 (4.9) |
| **Other relatives will come to Egypt** | Yes  No | 335 (63.1)  196 (36.9) |
| **Total** 531 | | |

**TableA-2:** Health conditions of family members

|  | **Labels** | **N (%)** | **Mean ± SD** |
| --- | --- | --- | --- |
| **Any family member suffering** | | |  |
| Diabetes | Yes  No | 175 (33.0)  356 (67.0) | 1.29 ± 0.59 |
| High blood pressure | Yes  No | 218 (41.1)  313 (58.9) | 1.32 ± 0.66 |
| Heart diseases | Yes  No | 59 (11.1)  472 (88.9) | 1.05 ± 0.22 |
| Diseases of the spine | Yes  No | 120 (22.6)  411 (77.4) | 1.16 ±0.43 |
| Joint stiffness | Yes  No | 184 (34.7)  347 (65.3) | 1.12 ± 0.39 |
| Allergy | Yes  No | 170 (32.0)  361 (68.0) | 1.34 ±0.77 |
| Movement disability | Yes  No | 47 (8.9)  484 (91.1) | 1.09 ± 0.35 |
| Intellectual disability | Yes  No | 21 (4.0)  510(96.0) | 1.14 ± 0.36 |
| Vision disability | Yes  No | 59 (11.1)  472 (88.9) | 1.51 ± 0.86 |
| Hearing disability | Yes  No | 24 (4.5)  507 (95.5) | 1.04 ± 0.20 |
| Other chronic diseases | Yes  No | 105 (19.8)  426 (80.2) | 1.23 ± 0.62 |
| Anemia or malnutrition | Yes  No | 107 (20.2)  424 (79.8) | 1.30 ± 0.73 |
| Need monthly medication | Yes  No | 338 (63.7)  193 (36.3) | 1.53 ± 0.85 |
| Total 531 (100) | | | |

**Table A-3**: living conditions of Sudanese refugees in Egypt.

| **Characteristics** | labels | N(%) |
| --- | --- | --- |
| **Kind of house in Egypt** | Rented apartment  With friends or family  Other | 287 (54.0)  225 (42.4)  19 (3.6) |
| **Live in shared apartment with others** | Yes (Sudanese family)  No  Don’t report | 125 (23.5)  178 (33.5)  228 (42.9) |
| **Have relatives or acquaintances residing in Egypt before the crisis** | Yes  No | 305 (57.4)  226 (42.6) |
| **Employment status in Sudan** | Were working  Unemployed and looking for job  Outside labour force | 333(62.7)  25 (4.7)  173 (32.6) |
| **Employment status in Egypt** | Employed  Unemployed and looking for job  Intends to work  Outside labour force | 45 (8.5)  179 (33.7)  189 (35.6)  118 (22.2) |
| **Had any family member currently working** | Yes  No | 27 (5.1)  504 (94.9) |
| **Main source of family expenditures in Egypt** | Income from work  Money saved from Sudan  Transfers from abroad  Subsidies  Other | 48 (9.0)  186 (35.0)  73 (13.7)  212 (39.9)  12 (2.3) |
| **Main source of family’s food expenditure** | Buy food cash with my money  Subsidies  Other | 316 (59.5)  212 (39.9)  3 (0.6) |
| **Total** |  | 531 (100) |

**Table A-4:**Aid and challenges facing Sudanese refugees

|  | Labels |  |
| --- | --- | --- |
| **Receiving financial, in kind or services** | Yes, financial aid  Yes, in kind assistance  Yes, financial aid & in kind assistance  No | 176 (33.1)  125 (23.5)  84 (15.8)  146 (27.5) |
| **Total value of financial aid that the family received** | 2335.68±2792 | |
| **The assistance suffices the basic needs** | Sufficient  Not enough  Not enough t all  Not applicable | 28 (5.2)  127 (23.9)  230 (43.3)  146 (27.5) |
| **Got an instant card from WFP or UNICEF** | Yes  No | 270 (50.8)  261 (49.2) |
| **Know about E wallet Applications** | Yes  No | 152 (28.6)  379 (71.4) |
| Most important problems | Need house |  |
